# First arrived takes all: inhibitory priority effects dominate competition between co-infecting *Borrelia burgdorferi* strains

**DOI:** 10.1186/s12866-015-0381-0

**Published:** 2015-03-07

**Authors:** Godefroy Devevey, Trang Dang, Christopher J Graves, Sarah Murray, Dustin Brisson

**Affiliations:** Department of Biology, Leidy Laboratories, University of Pennsylvania, Hamilton Walk, Philadelphia, PA 19104 USA; Institute of Evolutionary Biology, Ashworth Laboratories, University of Edinburgh, King’s Building Campus, EH9 3JT Edinburgh, UK; Department of Integrative Biology, Oregon State University, Corvallis, OR 97331 USA; Department of Ecology and Evolutionary Biology, Walter Hall, Brown University, 80 Waterman Street, Providence, RI 02912 USA

**Keywords:** *Borrelia burdgorferi*, OspC, Priority effect, Transmission, Coinfection, Strain competition, Apparent competition

## Abstract

**Background:**

Within-host microbial communities and interactions among microbes are increasingly recognized as important factors influencing host health and pathogen transmission. The microbial community associated with a host is indeed influenced by a complex network of direct and indirect interactions between the host and the lineages of microbes it harbors, but the mechanisms are rarely established. We investigated the within-host interactions among strains of *Borrelia burgdorferi*, the causative agent of Lyme disease, using experimental infections in mice. We used a fully crossed-design with three distinct strains, each group of hosts receiving two sequential inoculations. We used data from these experimental infections to assess the effect of coinfection on bacterial dissemination and fitness (by measuring the transmission of bacteria to xenodiagnostic ticks) as well as the effect of coinfection on host immune response compared to single infection.

**Results:**

The infection and transmission data strongly indicate a competitive interaction among *B. burgdorferi* strains within a host in which the order of appearance of the strain is the main determinant of the competitive outcome. This pattern is well described by the classic priority effect in the ecological literature. In all cases, the primary strain a mouse was infected with had an absolute fitness advantage primarily since it was transmitted an order of magnitude more than the secondary strain. The mechanism of exclusion of the secondary strain is an inhibition of the colonization of mouse tissues, even though 29% of mice showed some evidence of infection by secondary strain. Contrary to expectation, the strong and specific adaptive immune response evoked against the primary strain was not followed by production of immunoglobulins after the inoculation of the secondary strain, neither against strain-specific antigen nor against antigens common to all strains. Hence, the data do not support a major role of the immune response in the observed priority effect.

**Conclusion:**

The strong inhibitory priority effect is a dominant mechanism underlying competition for transmission between coinfecting *B. burgdorferi* strains, most likely through resource exploitation. The observed priority effect could shape bacterial diversity in nature, with consequences in epidemiology and evolution of the disease.

**Electronic supplementary material:**

The online version of this article (doi:10.1186/s12866-015-0381-0) contains supplementary material, which is available to authorized users.

## Background

Recent advances have shown that the community of microbial species within a host has substantial and direct effects on host fitness and trophic interactions in the host community [1-5]. When multiple species of microparasites infect the same host, the interactions among parasites can exacerbate the effects of the microbial communities on host fitness [6-8] and select for mechanisms that alter the epidemiology of the parasites (Devevey G, Knowles SCL, Withenshaw S, Petchey OL, Pedersen AB, Fenton A. Population-level consequences of interactions at the individual-host level within a co-circulating community of pathogens, Submitted). Investigations into the pattern and mechanisms involved in the interactions among microbes within a host are important as the overwhelming majority of vertebrates in nature are infected by multiple microbial species simultaneously, often with multiple lineages of the same species [9-12]. Strains of the same species are likely to occupy the same ecological niche and are therefore predicted to have more intense competitive interactions affecting their respective fitness; in addition strains can also facilitate each other, maximizing overall infection and the transmission [10,13]. Here we use *Borrelia burgdorferi*, the bacterium responsible for human Lyme disease, as a model to investigate patterns and mechanisms of ecological interactions among microbial strains of the same species and to investigate the effects of these interactions on microbial fitness.

Ixodid ticks, the genus that carries and transmits *B. burgdorferi* among vertebrate hosts, can acquire multiple *B. burgdorferi* strains by feeding on a single infected host [12,14-17]. In geographic regions where *B. burgdorferi* is prevalent, individual white footed mice - *Peromyscus leucopus*, the main host of the bacterium in the wild - are often infected with multiple strains of *B. burgdorferi* due to either the multiplicity of infection within individual feeding ticks [14,18] or to the large number of infectious tick bites each individual vertebrate incurs over its lifetime [19,20]. Thus, multiple *B. burgdorferi* strains commonly infect individual vertebrate hosts and individual tick vectors, providing the opportunity for both direct competition (mediated by interference) and indirect interactions (mediated by resource exploitation and host defenses).

Strains of *B. burgdorferi* are transmitted to ticks feeding on *Peromyscus leucopus* mice at much higher rates when the mouse is only infected with a single strain than when the mouse is infected by two or more strains [18,21]. These results are consistent in both experimentally and naturally infected mice, indicating that the fitness of each strain is compromised by co-infections with other strains. While these data suggest that *B. burgdorferi* strains interact competitively within a host, this hypothesis has not been experimentally investigated and the types of competitive interactions among strains within hosts are not known. Any of the three competition types (i.e. interference, exploitation, or apparent [22]) could be operating in this system. For example, a primary strain could actively prevent the dissemination of a secondary strain in adequate sites, reducing overall transmission success of the secondary strains to feeding ticks. Alternatively, a strain could exploit and deplete a resource at the expense of others thanks to a more efficient exploitation or early segregation of the resource. Lastly, a strain could prime the host immune response to target an incoming strain in a process resembling apparent competition [9]. These processes are not mutually exclusive and each mechanism could affect the competitive outcome among infecting strains.

In this study we looked for empirical evidence of competition among three coinfecting strains of *B. burgdorferi,* assessed the ecological patterns of the competition, and investigated a potential molecular mechanism affecting competition. The wild bacteria *B. burgdorferi* is particularly variable at the genetic locus coding for the outer surface protein C (OspC) [23]; OspC is critical for the establishment of the bacteria in early stages of mammalian infection [24] even though the exact mechanism of action is still debated [25-27] and is known to trigger a strong immune response [28-30]. Some strains are more commonly found in certain host species than others [18]. We used the *ospC* strains A, K, and N, which are infectious to humans [28]. Mice received two sequential inoculations to mimic the timing in which hosts can be exposed to *B. burgdorferi*-infected ticks in nature [20,29]. The fully crossed-design created three groups of mice exposed to homologous infections (A-A, K-K, N-N), and six groups of mice exposed to heterologous infections (A-K, A-N, K-A, K-N, N-A, N-K). We investigated the fitness of each strain as the rate of transmission of that strain to feeding ticks four times during the 90-day experiment (twice before and twice after the secondary inoculation). Additionally, we investigated if strains interfered with the colonization of disseminated sites within hosts by testing for the presence of each strain in four tissues of each host. These data allow us to assess the competitive hierarchy among strains, the effect of a secondary infection on the fitness of a disseminated strain, and the ability of each strain to colonize a previously infected host. To test if the observed competition was mediated by the adaptive immune system of the host, we also measured the immunoglobulin G produced against strain-specific antigen (OspC), against the flagellin protein (Fla) common to all *B. burgdorferi* strains, and against all antigens (total IgG).

## Methods

### *Borrelia burgdorferi* and mice

**Figure 1:**
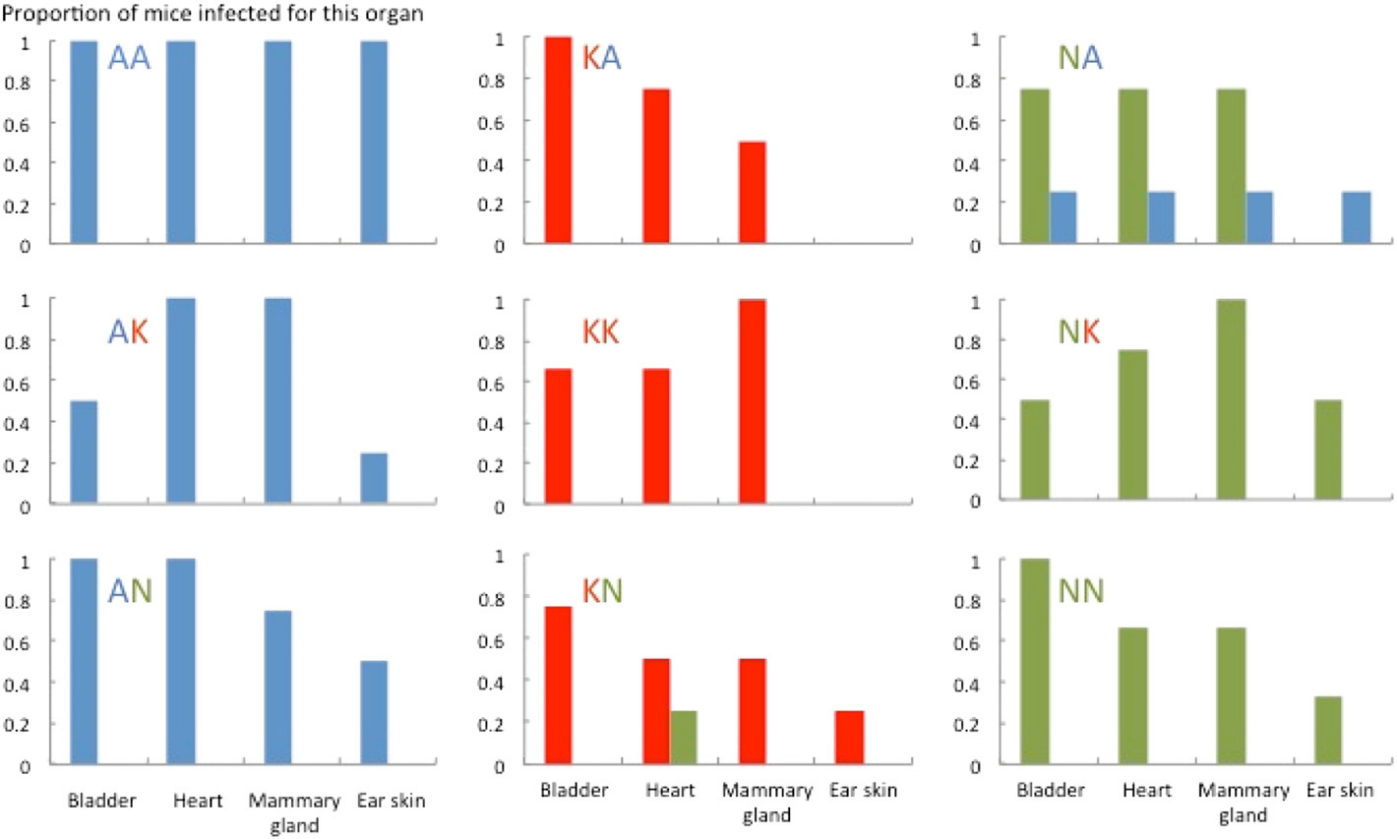
**Infection in mouse tissues at Day 90 was dominated by the primary strain.** Few tissue samples tested positive for the secondary strains. Ear tissue was less frequently infected than the other organs. n = 4 mice per group except in groups KK and NN where n = 3 mice.

We used three *Borrelia burgdorferi* sensu stricto isolates that differed at their *ospC* locus to experimentally infect 36 three-month old female C3H/HeJ mice (Charles River). The *B. burgdorferi* isolates 97–064 (strain A), 97–010 (strain K), and 931222 (strain N) were obtained through the Center for Disease Control as part of the standard patient care, which did not require ethical approval. Each strain (passage 3–5) was grown in BSK-II complete medium [30] at 35°C, washed three times with phosphate buffer saline (PBS) and concentrations were equalized after cell counting by hemocytometer, prior to subcutaneous injection of 5*10^4^ cells in 200 μl of PBS. Mice were randomly assigned to a co-infection treatment (4 mice per treatment) after acclimation to the animal facility for one week. Mice were randomly paired and kept two per cage. Two individuals in groups KK and NN died for unknown reasons during the 90-day course of the experiment. Mice in the homologous co-infection treatment groups were infected with same strain on days 0 and day 35 (d35) of the experiment (A-A, K-K, N-N). Mice in the heterologous co-infection treatments groups were infected by two different strains during the experiment (A-K, K-A; A-N, N-A; K-N, N-K). At day 90, mice were sacrificed by CO_2_ inhalation and blood was collected by cardiac puncture. Tissue samples from the ear, the bladder, the heart, and the mammary glands were extracted and frozen for subsequent analysis of tissue tropism of each strain (Figure 1). The animal experimentation protocol was approved by the University of Pennsylvania IACUC (801614).

### Infection status

The infection status of each mouse was determined using nested PCR of the *ospC* gene of mouse tissue samples and xenodiagnostic ticks. Xenodiagnosis, allowing uninfected larval ticks to feed on a host, is a traditional method of diagnosis for vector-borne diseases and can be used to estimate the transmission efficiency of *B. burgdorferi* from an infected mouse to naïve larval ticks. Xenodiagnostic larval ticks (*c.* 50 per mouse) were placed on each mouse on days 11, 30, 46, and 65 and allowed to feed to repletion [31]. Engorged larval ticks were kept individually in microtubes containing a 1-cm^2^ square of moistened blotting paper until they molted to the nymphal stage. DNA from 40 whole nymphal ticks (up to 10 per xenodiagnosis) or 25 μg of each mouse tissue was extracted using the DNeasy Blood and Tissue kit (Qiagen, Hilden, Germany). The presence of *B. burgdorferi* in each sample was detected by nested PCR of the *ospC* gene using two sets of specifically designed primers that targeted conserved regions for all our strains (based on 65 sequences from Genbank). The first PCR reaction amplified a 1087 bp fragment using primers OC-368 (forward: 5’-ATAAACGCCAATTTCTCTAATTCTTC-3’) and OC693 (reverse: 5’-GACTTTATTTTTCCAGTTACTTTTTT-3’). The second PCR reaction amplified a 657 bp fragment using primers OC4 (5’-GAAAAAGAATACATTAAGTG-3’) and OC643 (reverse: 5’-TAATTAAGGTTTTTTTGGA-3’). The first PCR reaction contained 4 μl of the DNA extraction, 2.4 mM MgCl2, 0.2 mM dNTP, 0.5 μM of each primer, 1X Buffer, and 1 unit of recombinant Taq (Invitrogen, LifeTechnologies, Carlsbad CA, USA) in a total volume of 50 μl. After an initial denaturation at 95°C for 1 min, the mixture was run for 30 cycles at 95°C for 40 sec, 54°C for 35 sec, and 72°C for 60 sec. The conditions for the second PCR reaction were the same as above except that 2 μl of product from the first PCR reaction was used as template, annealing temperature was 53°C, and the reaction was run for 40 cycles. The resulting products were run on a 1% ethidium bromide agarose gel to assess the presence of *B. burgdorferi*. All samples were tested by PCR two times to protect against false positive and false negative assignments.

### Samples with multiple strains

PCR products were digested with allele-specific restriction enzymes to identify the *ospC* alleles present in each sample. The *ospC* alleles A, K, and N can be cleaved by restriction enzymes Bgl II (Fermentas Life Sciences), Pst I, and Sac I (New England Biolabs Inc.), respectively. Digestion products were run on 2% ethidium bromide agarose gel in parallel with control PCR products (without restriction enzyme).

### Immune response

The total titer of circulating immunoglobulin-G (IgG) and the titer of IgG specific for *B. burgdorferi* flagellin, OspC-A, OspC-K, or OspC-N was measured by ELISA. Blood was collected by submandibular puncture from each mouse on days 11, 30, 46, and 65, centrifuged at 2500 rpm for 10 min, and the sera from each sample was collected and frozen at −20°C. Total IgG titers were measured using the Mouse total IgG ELISA kit Ready-SET-Go! (eBioscience, San Diego CA, USA) and following the protocol of the manufacturer. The titers of antibodies against individual *B. burgdorferi* proteins were measured by coating NUNC maxisorbent 96-well plates with the *B. burgdorferi* protein overnight. Recombinant *B. burgdorferi* OspC proteins were produced as described in [26] and were diluted at 10 ng/μl in PBS. Recombinant flagellin was purchased (Prospec-Tany Technogene Ltd., Nest Ziona, Israel) and diluted to 2 ng/μl in 5 M urea. After incubation, each well was blocked with 2% BSA for two hours and incubated with serum diluted 1:110 in 2% BSA for each OspC protein or 1:1000 in PBS for flagellin. Each well was then incubated for 45 min with the secondary antibody (alkaline phosphatase linked anti-*Mus musculus* IgG goat antibody (Sigma, St-Louis MO) diluted at 1:5000). All wells were washed three times with PBS-Tween 0.1% between each step. The color reagent p-Nitrophenyl phosphate (Sigma, St-Louis MO) was added and the kinetic phosphatase reaction was monitored at 405 nm for 30 minutes (one reading every minute).

### General statistical methods

Differences in the infection of mouse tissues and the mouse-to-tick transmission were modeled using generalized linear mixed effects models (GLMMs) with binomial errors while differences in antibody titers were modeled using GLMMs with normal error terms. The models used to test the effect of the order of inoculation or strain identity on the response variables included strain identity (A, K, or N), the order of inoculation (primary or secondary), and the date of xenodiagnoses (day 11, 30, 46, or 65) as fixed factors, all possible 2-way and 3-way interaction terms, and mouse identity (N = 34) as a random factor. The effect of the order of inoculation on mouse-to-tick transmission during the last two transmission events when both strains were present in the mouse (d46 and d65) also included ticks nested within mouse as a random factor. The statistical significance of fixed factors and interactions among fixed factors for each model were determined using likelihood ratio tests. Additionally, the most parsimonious models were selected using the AIC model selection approach. Analyses were done using the lme4 package [32] in R (version 2.10). Final models are exposed in Additional file 1.

## Results

### Primary strain infection and transmission

Evidence of infection by the primary strain was present in both the tissue samples and the xenodiagnostic ticks (i.e. ticks that fed on mice to test for infection) from all mice. After sacrifice, the primary strain was found in at least one tissue from all mice although most mice showed evidence of the primary strain in multiple tissues (average of 2.9 infected tissues per host). Evidence of active infection was least prevalent in ear tissue (11 of 34 mice) but common in the heart, bladder and mammary gland (Likelihood ratio test for “tissue” factor: Δdev = 25.78, Δdf = 3, p < 0.001; Figure 1). The identity of the strain affected the probability of detecting infection in tissues (Δdev = 16.38, Δdf = 2, p < 0.001) as strain A was more commonly detected than strains K or N (Additional file 1: Table S1).

**Figure 2:**
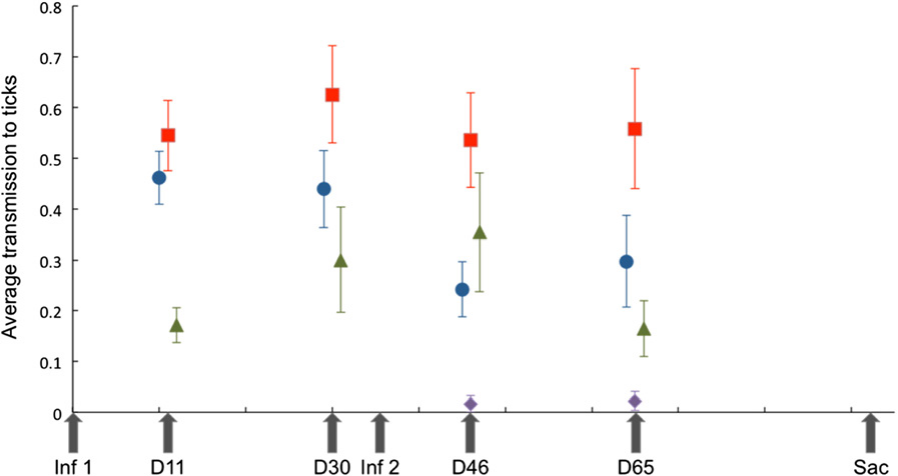
**Average mouse-to-tick transmission rates (± standard error) of the primary strain.** Strain A - blue circles; strain K - red squares; strain N - green triangles. The mouse-to-tick transmission rate of the secondary strain was pooled for the three strain types (purple diamonds). *Inf 1* - day of inoculation with primary strain; *Inf 2* - day of inoculation with secondary strain. D11, D30, D46, D65 refer to the days of blood sampling and xenodiagnosis. Sac = sacrifice for biopsy of organs (D90).

The primary strain was also frequently detected in xenodiagnostic ticks that had taken their larval blood meal from the experimental mice (Figure 2) (Additional file 1: Table S2). Of the 453 ticks that tested positive for *B. burgdorferi,* the primary strain was present in 446. The identity of the strain affected the rate at which the strain was transmitted to feeding ticks (Δ dev = 13.63, Δdf = 2, p = 0.001), with strain K being transmitted at the highest rate followed by strain A (Figure 2). The rate of transmission of the primary strain to feeding ticks varied among the four xenodiagnostic time points (Δdev = 11.64, Δdf = 3, p = 0.009), although the pattern of temporal variation differed significantly among strains (Δdev = 33.67, Δdf = 6, p < 0.001). While strain K was transmitted to feeding ticks at a consistently high rate throughout the experiment, the transmission rate of strain A decreased over time, and the transmission rate of strain N peaked in the middle time points.

### Secondary strain infection and transmission

The secondary strain – the second strain the mice were exposed to – was detected in only seven of the 24 mice that were challenged with two different strains. Of these seven mice, only three had evidence of infection with the secondary strain in tissue samples; strain A was found in the ear, heart, and mammary tissues of one mouse and in the bladder of another mouse, and strain N was found in the heart of a KN mouse (Additional file 1: Figure S1). This mouse, like four others, showed evidence of secondary strain infection only by one to two positive xenodiagnostic ticks. Strain K was transmitted from three mice, strain A from one mouse, and strain N from one mouse. Neither strain identity, time of xenodiagnosis, nor any interaction term significantly affected the mouse-to-tick transmission rate of the secondary strain. Interestingly, the identity of the secondary strain did not influence the mouse-to-tick transmission of the primary strain (Δdev = 26.26, Δdf = 24, p = 0.34) (Additional file 1: Table S1). Since only seven ticks carried the secondary strain (from mice in groups AK, KA, KN, NK), we lacked power to statistically test the effect of the primary strain on the transmission of the secondary strain, but we observed transmission of the secondary strain with each of our primary strains.

For mice inoculated with multiple *B. burgdorferi* strains, mouse-to-tick transmission of the primary strain was an order of magnitude higher than that of the secondary strain during the last two transmission events when both strains were present in the mouse (Table 1). Thus, the order of inoculation of the strains had a significant effect on the probability of transmission to mice (Δ dev = 196.2, Δ df = 1, p < 0.001). The log-odds ratio of the contrast between the secondary and the primary strain was −4.110 (s.e. = 0.480; Additional file 1: Table S3). That is, the probability of transmission to feeding larvae was 31-fold greater when a strain is inoculated first than when it was inoculated second.

**Table 1:** **Mouse-to-tick transmission was more successful for the primary strain than the secondary strain**

	**D46**		**D65**	
**Strain**	**Primary**	**Secondary**	**Primary**	**Secondary**
A	100% (21)	0%	90% (9)	10% (1)
K	94% (46)	6% (3)	94% (32)	6% (2)
N	97% (29)	3% (1)	100% (7)	0%

Transmission success was calculated as the proportion of ticks infected with strain 1 (or strain 2) relative to the total number of infected ticks. The number of ticks infected with each strain are in brackets.

### Immune response

**Figure 3:**
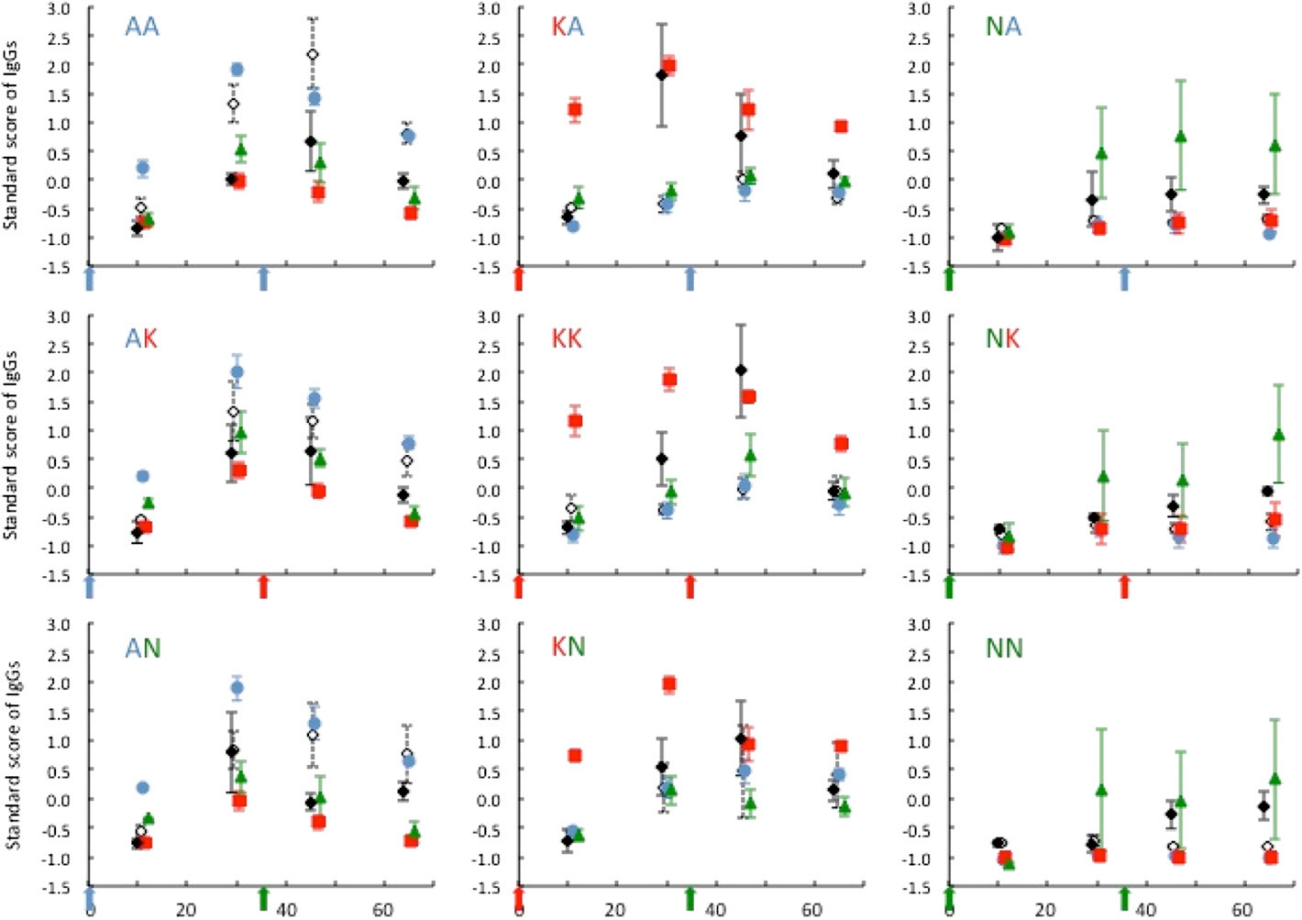
**The primary strain induced a strong strain-specific adaptive immune response.** The secondary strain did not alter immune response. Each panel shows one of nine co-infection treatments. Antibody profiles are similar within columns, which share the primary strain, but differ among rows, which share the secondary strain. This pattern indicates that the primary strain dominated the antibody response and that antibody profiles differed among primary strains (Additional file 1: Table S6 and S7). Arrows indicate the timing of the primary and secondary inoculation. Shown are the mean antibody titers (± S.E.) for total IgG (black diamond), anti-flagellin IgG (white diamond), anti-OspCA IgG (blue circle), anti-OspCK (red square), and anti-OspCN (green triangle). To facilitate viewing, absolute differences in scale among the five antibody variables were removed by standardizing them to z-scores (mean = 0, stdev = 1).

The primary strain induced a strong immune response as measured by total immunoglobulin G (IgG) production as well as a strong specific immune response against flagellin and against the OspC genotype expressed by the primary strain (Figure 3). The anti-OspC response induced against the primary strain had low cross-reactivity with the OspC antigen of the secondary strains. In other words, the anti-OspC antibodies induced by strain A infection had low affinity for OspC type K nor type N proteins and vice versa. The inoculation of the secondary strain did not affect the total IgG level, the anti-Fla response, nor the specific anti-OspC response targeting either the primary or the secondary strain, even in the mice with conclusive evidence of infection by the secondary strain.

We used principal component analysis (PCA) to characterize the antibody profiles of the mice as the five antibody response variables (OspCA-IgG, OspCK-IgG, OspCN-IgG, Fla-IgG, and total IgG) were strongly and positively correlated (Additional file 1: Table S4). The first principal component (PC1) captured 44.5% of the total variance in the data (Additional file 1: Table S5) and represented the overall IgG response as the five antibody variables were all positively correlated with this component. The second principal component (PC2) explained 22.5% of the total variance (Additional file 1: Table S5) and represented a contrast between high levels of total IgG and OspCK-IgG and low levels of Fla-IgG and OspCA-IgG. Thus PC2 can be considered as a measure of specificity because individuals that have a strong anti-OspCA response have a weak anti-OspCK response and vice versa. The ability of our PCA to capture the relevant variation in the antibody profiles is shown by the scatterplot of PC1 versus PC2, which causes mice to cluster according to the primary strain and not according to the secondary strain (Additional file 1: Figure S2). The overall IgG response depended on the primary strain (Δdev = 30.70, Δ df = 2, p < 0.001), and the day of blood sampling (Δdev = 5.72, Δdf =1, p =0.017), but was independent of the secondary strain (Δdev = 0.84, Δdf = 2, p = 0.66; Additional file 1: Table S6). Similarly, only the primary strain affected the OspC-specific immune response (Δdev = 52.92, Δdf = 2, p < 0.001) while the day of blood sampling and the secondary strain had no effect (Δdev = 2.15, Δdf = 3, p = 0.542; Additional file 1: Table S7).

## Discussion

### Inhibitory priority effect

Interactions among co-infecting microbes in a host can alter the within-host microbial community and ultimately influence the diversity and population dynamics of microbes in nature. We used experimental infections to determine the competitive interactions among strains of *Borrelia burgdorferi*, the pathogenic bacterium that causes human Lyme disease. We detected the presence of strong, inhibitory competitive interactions among strains of *B. burgdorferi* within a host. The order in which strains were inoculated into the mouse was the dominant factor in the competitive outcome among strains. In all cases, the first strain to infect had a strong fitness advantage primarily because the colonization of mouse tissues by subsequent strains was inhibited. Although the strains differed in their rates of transmission in the absence of competition, indicating a potential competitive hierarchy, all strains were excluded when secondary in infection order regardless of the primary and secondary strain identity, suggesting the absence of a competitive hierarchy in sequential infections. The primary strain evoked a strong and specific adaptive immune response, whereas there was no change in antibody level following the secondary inoculation contrary to expectation of activation of a memory immune response. Overall, the data do not support a major role of apparent competition due to the adaptive immune response mediated by IgG in the observed priority effect.

Evidence of infection by the secondary strain was rare (Figure 1; Table 1) suggesting that the primary infection either reduced the probability of infection by the secondary strain or limited the population size of the secondary strain within the host. The data presented are consistent with the latter hypothesis as strains with small population sizes are difficult to detect in host tissues by PCR and would rarely be transmitted to feeding ticks [33,34].

Indirect interactions among strains mediated by the host immune system were not readily apparent from the data. All mice mounted a vigorous immune response against the initial strain, which could have been protective against subsequent *B. burgdorferi* infections [31,35-39]. However, the hypothesis that initial infections reduce the probability of infection by subsequent strains, similar to expectations from vaccination, is not congruous. It seems unlikely that previously induced antibodies against other *Borrelia* specific antigens, such as the antigenic proteins Outer surface protein A (OspA) or OspC, can explain the observed priority effect data for four reasons. First, the exclusion of the secondary strain occurred without increasing antibody titers following the inoculation of the secondary strain as would be expected if memory antibodies were involved. If memory antibodies were activated, they would trigger the proliferation of B cells that would then produce large amount of antibodies [40]. In contrast, the constant level of antibodies in our data suggests an immunosuppressive effect by the primary strain [41]. Second, at least 29% of mice were infected by the secondary strain but at very low levels. Vaccination effect would result in less mice infected with the second strain and would affect the transmission of the bacteria to ticks of all strains independently of the order of infection, which is not what we observed in mice successfully infected by the secondary strain [31]. Third, bacteria in culture express different phenotypes, some expressing OspC, others expressing other proteins [42]. If the rejection of the secondary strain was due to a specific antigen recognition, a proportion of the inoculated bacteria would be immune to the specific antibodies and would be able to disperse. Lastly, it is unlikely that the titer of antibodies from the first immune response was at saturation as mice primed with the N strain had significantly lower antibody titers than those initially infected by strains A or K yet still showed no increase when challenged by a second strain (Figure 3). Thus, the mechanism leading to the observed priority effect pattern is likely caused by direct or indirect exclusion (interference or resource competition) of the second strain from colonizing disseminated sites in the host. Future investigations using serial sampling of disseminated sites are necessary to observe direct interactions among strains.

### Ecological consequences of priority effect

The strength of the observed priority effect could, repeated over several years in a homogenous environment, reduce the diversity of *B. burgdorferi* strains in nature and rapidly lead to a uniform population. However, the diversity of *B. burgdorferi* strains remains high in many geographic areas and even within wild mice [12,14,17,18]. High strain diversity within wild mice has been observed in geographic regions where both tick vectors and *B. burgdorferi* are highly prevalent and mice are fed upon by tens of infected ticks, many carrying multiple strains, within a period of 30 days [20,29,43]. Thus, the wild mice in these studies were challenged by multiple strains nearly simultaneously, which would exclude the possibility of detecting a priority effect.

Competitive exclusion due to the priority effect mechanism may have an impact in geographic areas where *B. burgdorferi* and ticks are present but at low prevalence, such as areas where Lyme disease is spreading [44-47]. In these areas, the time between infectious tick bites can be long resulting in competitive exclusion of the latter strains and dramatic fitness benefits to the most prevalent strains. This effect has the potential to inhibit or prevent the invasion of subsequent strains into a geographic region. Additionally, in many areas the season in which ticks are active in extends over several months such that strains in late-season feeding ticks that take their blood meal at the end of the season will have lower success than those from early-season feeding ticks, even in highly endemic regions [45,48,49]. This scenario suggests that bacteria face strong selective pressures for the emergence of mechanisms to secure their transmission such as vector behavior manipulation for early or synchronous host seeking.

## Conclusions

The strong inhibitory priority effect is the dominant mechanism controlling the competitive outcome among *B. burgdorferi* strains in a sequentially challenged vertebrate host. This inhibitory priority effect occurs regardless of the fitness differences among strains in the absence of competition. Although the molecular mechanism leading to the observed priority effect could not be precisely determined with the current experiments, it is effective within 10 days of the secondary inoculation and results in the inhibition of dissemination or colonization of the host. The priority effect has the potential to limit or reduce the diversity of strains in nature, especially in low prevalence areas where sequential challenges are likely. Expansion of *B. burgdorferi* into new geographic areas or changes in tick questing behaviors in response to climate change may increase the impact of the priority effect on competitive interactions among *B. burgdorferi* strains and cause the most common *B. burgdorferi* lineages, which are also the most infectious in humans [28], to become even more prevalent.

## Additional file

Additional file 1: Figure S1. Chart summarizing the diagnostic of infection by first and secondary strain for each mouse. **Figure S2.** The immune response depends on the primary strain. **Table S1.** Organs were infected by the primary inoculated strain, though strains did not infect at the same rate and some organs were more susceptible to infection, without interactions. **Table S2.** Mouse-to-tick transmission of primary strain depended on the identity of the primary strain, the day of xenodiagnoses (age of infection), and the strain:day interaction. **Table S3.** Among heterologous mice at D46 and D65, mouse-to-tick transmission depended on the order of inoculation (primary/secondary), strain identity (A, K, and N), the day of xenodiagnoses (age of infection), and the strain:day interaction. **Table S4.** The titers of IgG were correlated. **Table S5.** The results from the principal component analysis (PCA). **Table S6.** The strength of the immune response, summarized by PC1, depended on the day of blood sampling and the primary strain, but was independent of the secondary strain. **Table S7.** The specific adaptive immune response, summarized by PC2, depended on the primary inoculated strain and the date considered, regardless of the secondary strain.

## References

[1] Backhed F, Ley RE, Sonnenburg JL, Peterson DA, Gordon JI (2005). Host-bacterial mutualism in the human intestine. Science.

[2] Foxman B, Goldberg D, Murdock C, Xi C, Gilsdorf JR (2008). Conceptualizing human microbiota: from multicelled organ to ecological community. Interdiscip Perspect Infect Dis..

[3] Ley RE, Peterson DA, Gordon JI (2006). Ecological and evolutionary forces shaping microbial diversity in the human intestine. Cell.

[4] McDermott AJ, Huffnagle GB (2014). The microbiome and regulation of mucosal immunity. Immunology.

[5] Thomas LV, Ockhuizen T, Suzuki K (2014). Exploring the influence of the gut microbiota and probiotics on health: a symposium report. Br J Nutr..

[6] Mideo N (2009). Parasite adaptations to within-host competition. Trends Parasitol.

[7] Alizon S, van Baalen M (2008). Multiple infections, immune dynamics, and the evolution of virulence. Am Nat.

[8] Cox FEG (2001). Concomitant infections, parasites and immune responses. Parasitology..

[9] Pedersen AB, Fenton A (2007). Emphasizing the ecology in parasite community ecology. Trends Ecol Evol.

[10] Read AF, Taylor LH (2001). The ecology of genetically diverse infections. Science.

[11] Rigaud T, Perrot-Minnot M-J, Brown MJ (2010). Parasite and host assemblages: embracing the reality will improve our knowledge of parasite transmission and virulence. Proc R Soc B Biol Sci.

[12] Perez D, Kneubuehler Y, Rais O, Jouda F, Gern L (2011). Borrelia afzelii ospC genotype diversity in ixodes ricinus questing ticks and ticks from rodents in two Lyme borreliosis endemic areas: contribution of co-feeding ticks. Ticks Tick-Borne Dis.

[13] Taylor LH, Walliker D, Read AF (1997). Mixed-genotype infections of the rodent malaria Plasmodium chabaudi are more infectious to mosquitoes than single-genotype infections. Parasitology..

[14] Andersson M, Scherman K, Raberg L (2013). Multiple-strain infections of borrelia afzelii: a role for within-host interactions in the maintenance of antigenic diversity?. Am Nat.

[15] Perkins SE, Cattadori IM, Tagliapietra V, Rizzoli AP, Hudson PJ (2003). Empirical evidence for key hosts in persistence of a tick-borne disease. Int J Parasitol.

[16] Qiu WG, Dykhuizen DE, Acosta MS, Luft BJ (2002). Geographic uniformity of the Lyme disease spirochete (Borrelia burgdorferi) and its shared history with tick vector (Ixodes scapularis) in the northeastern United States. Genetics.

[17] Swanson KI, Norris DE (2008). Presence of multiple variants of Borrelia burgdorferi in the natural reservoir Peromyscus leucopus throughout a transmission season. Vector Borne Zoonotic Dis.

[18] Brisson D, Dykhuizen DE (2004). OspC diversity in borrelia burgdorferi: different hosts are different niches. Genetics.

[19] Brunner JL, Ostfeld RS (2008). Multiple causes of variable tick burdens on small-mammal hosts. Ecology.

[20] Devevey G, Brisson D (2012). The effect of spatial heterogenity on the aggregation of ticks on white-footed mice. Parasitology.

[21] Derdakova M, Dudioak V, Brei B, Brownstein JS, Schwartz I, Fish D (2004). Interaction and transmission of two Borrelia burgdorferi sensu stricto strains in a tick-rodent maintenance system. Appl Environ Microbiol.

[22] Begon M, Townsend CR, Harper JL (2006). Ecology: from individuals to ecosystems.

[23] Wang IN, Dykhuizen DE, Qin WG, Dunn JJ, Bosler EM, Luft BJ (1999). Genetic diversity of ospC in a local population of Borrelia burgdorferi sensu stricto. Genetics.

[24] Tilly K, Krum JG, Bestor A, Jewett MW, Grimm D, Bueschel D (2006). Borrelia burgdorferi OspC protein required exclusively in a crucial early stage of mammalian infection. Infect Immun.

[25] Earnhart CG, Rhodes DVL, Smith AA, Yang X, Tegels B, Carlyon JA (2014). Assessment of the potential contribution of the highly conserved C-terminal motif (C10)of Borrelia burgdorferi outer surface protein C in transmission and infectivity. Pathog Dis.

[26] Oender O, Humphrey PT, McOmber B, Korobova F, Francella N, Greenbaum DC (2012). OspC is potent plasminogen receptor on surface of Borrelia burgdorferi. J Biol Chem.

[27] Pulzova L, Bhide M (2014). Outer surface proteins of borrelia: peerless immune evasion tools. Curr Protein Pept Sci.

[28] Dykhuizen DE, Brisson D, Sandigursky S, Wormser GP, Nowakowski J, Nadelman RB (2008). Short report: the propensity of different borrelia burgdorferi sensu stricto genotypes to cause disseminated infections in humans. Am J Trop Med Hyg.

[29] Ostfeld RS, Hazler KR, Cepeda OM (1996). Temporal and spatial dynamics of Ixodes scapularis (Acari: Ixodidae) in a rural landscape. J Med Entomol.

[30] Zuckert WR (2007). Laboratory maintenance of *Borrelia burgdorferi*. Curr Protocols Microbiol.

[31] Voordouw MJ, Tupper H, Onder O, Devevey G, Graves CJ, Kemps BD (2013). Reductions in human Lyme disease risk Due to the effects of oral vaccination on tick-to-mouse and mouse-to-tick transmission. Vector Borne Zoonotic Dis.

[32] Bates D, Maechler M, Bolker B, Walker S. lme4: Linear mixed-effects models using Eigen and S4. 2014; R package version 1.1-7.

[33] Aguero-Rosenfeld ME, Wang GQ, Schwartz I, Wormser GP (2005). Diagnosis of Lyme borreliosis. Clin Microbiol Rev.

[34] Brouqui P, Bacellar F, Baranton G, Birtles RJ, Bjoersdorff A, Blanco JR (2004). Guidelines for the diagnosis of tick-borne bacterial diseases in Europe. Clin Microbiol Infect.

[35] de Silva AM, Telford SR, Brunet LR, Barthold SW, Fikrig E (1996). Borrelia burgdorferi OspA is an arthropod-specific transmission-blocking Lyme disease vaccine. J Exp Med.

[36] Fikrig E, Barthold SW, Kantor FS, Flavell RA (1990). Protection of mice against the Lyme disease agent by immunizing with recombinant ospA. Science.

[37] Earnhart CG, Marconi RT (2007). An octavalent Lyme disease vaccine induces antibodies that recognize all incorporated OspC type-specific sequences. Hum Vaccin.

[38] Xu QL, McShan K, Liang FT (2008). Essential protective role attributed to the surface lipoproteins of Borrelia burgdorferi against innate defences. Mol Microbiol.

[39] Xu QL, Seemanapalli SV, McShan K, Liang FT (2006). Constitutive expression of outer surface protein C diminishes the ability of Borrelia burgdorferi to evade specific humoral immunity. Infect Immun.

[40] Janeway CA, Travers P, Walport M, Hunt S (1997). Immunobiology: the immune system in health and disease.

[41] Diterich I, Rauter C, Kirschning CJ, Hartung T (2003). Borrelia burgdorferi-induced tolerance as a model of persistence via immunosuppression. Infect Immun.

[42] Srivastava SY, de Silva AM (2008). Reciprocal expression of ospA and ospC in single cells of Borrelia burgdorferi. J Bacteriol.

[43] Ostfeld RS, Miller MC, Hazler KR (1996). Causes and consequences of tick (*Ixodes scapularis*) burdens on white-footed mice (*Peromyscus leucopus*). J Mammal.

[44] Khatchikian CE, Prusinski M, Stone M, Backenson PB, Wang I-N, Levy MZ (2012). Geographical and environmental factors driving the increase in the Lyme disease vector Ixodes scapularis. Ecosphere..

[45] Ogden NH, Bigras-Poulin M, Hanincova K, Maarouf A, O'Callaghan CJ, Kurtenbach K (2008). Projected effects of climate change on tick phenology and fitness of pathogens transmitted by the North American tick Ixodes scapularis. J Theor Biol.

[46] Nadolny RM, Wright CL, Hynes WL, Sonenshine DE, Gaff HD (2011). Ixodes affinis (Acari: Ixodidae) in southeastern Virginia and implications for the spread of Borrelia burgdorferi, the agent of Lyme disease. J Vector Ecol.

[47] Nadolny RM, Wright CL, Sonenshine DE, Hynes WL, Gaff HD (2014). Ticks and spotted fever group rickettsiae of southeastern Virginia. Ticks Tick Borne Dis.

[48] Ogden NH, Radojevic M, Wu X, Duvvuri VR, Leighton PA, Wu J (2014). Estimated effects of projected climate change on the basic reproductive number of the Lyme disease vector Ixodes scapularis. Environ Health Perspect.

[49] Simon JA, Marrotte RR, Desrosiers N, Fiset J, Gaitan J, Gonzalez A (2014). Climate change and habitat fragmentation drive the occurrence of Borrelia burgdorferi, the agent of Lyme disease, at the northeastern limit of its distribution. Evol Appl.

