# Supplementary material for: First arrived takes all: inhibitory priority effects dominate competition between co-infecting Borrelia burgdorferi strains
Source: BMC Microbiol. 2015 Mar 7;15:61. doi: 10.1186/s12866-015-0381-0 (PMC4359528; doi:10.1186/s12866-015-0381-0)
Supplement: Additional file 1: Figure S1. — Chart summarizing the diagnostic of infection by first and secondary strain for each mouse. Figure S2. The immune response depends on the primary strain. Table S1. Organs were infected by the primary inoculated strain, though strains did not infect at the same rate and some organs were more susceptible to infection, without interactions. Table S2. Mouse-to-tick transmission of primary strain depended on the identity of the primary strain, the day of xenodiagnoses (age of infection), and the strain:day interaction. Table S3. Among heterologous mice at D46 and D65, mouse-to-tick transmission depended on the order of inoculation (primary/secondary), strain identity (A, K, and N), the day of xenodiagnoses (age of infection), and the strain:day interaction. Table S4. The titers of IgG were correlated. Table S5. The results from the principal component analysis (PCA). Table S6. The strength of the immune response, summarized by PC1, depended on the day of blood sampling and the primary strain, but was independent of the secondary strain. Table S7. The specific adaptive immune response, summarized by PC2, depended on the primary inoculated strain and the date considered, regardless of the secondary strain. [file 12866_2015_381_MOESM1_ESM.docx]

First arrived takes all: Inhibitory priority effects dominate competition between co-infecting Borrelia burgdorferi strains

**ADDITIONAL FILE 1**

**Figures 1 and 2**

**Tables 1 to 5**

**
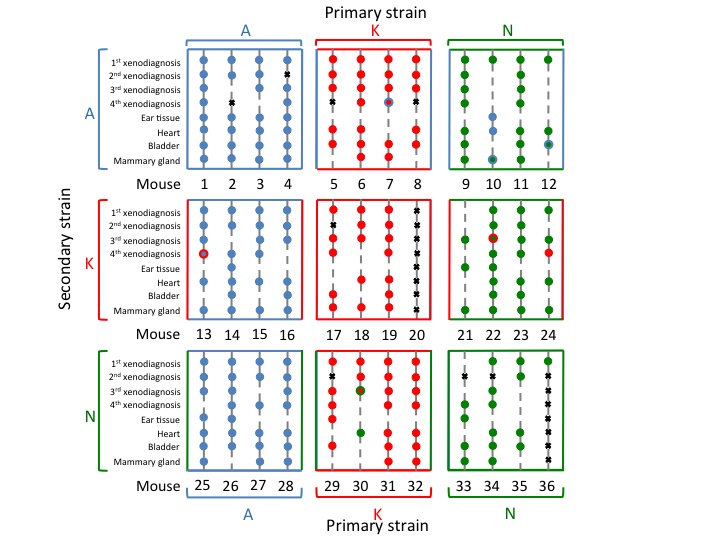
**

**Supplementary Figure 1.** Chart summarizing the diagnostic of infection by first and secondary strain for each mouse. Spots represent samples (ticks or organs) and are colored by the strain found (A: blue; K; Red; N: Green). Spots are bicolor if two strains were found in the sample. Spots are blank when diagnostic tests were negative for any strain, and crossed if no sample was available.


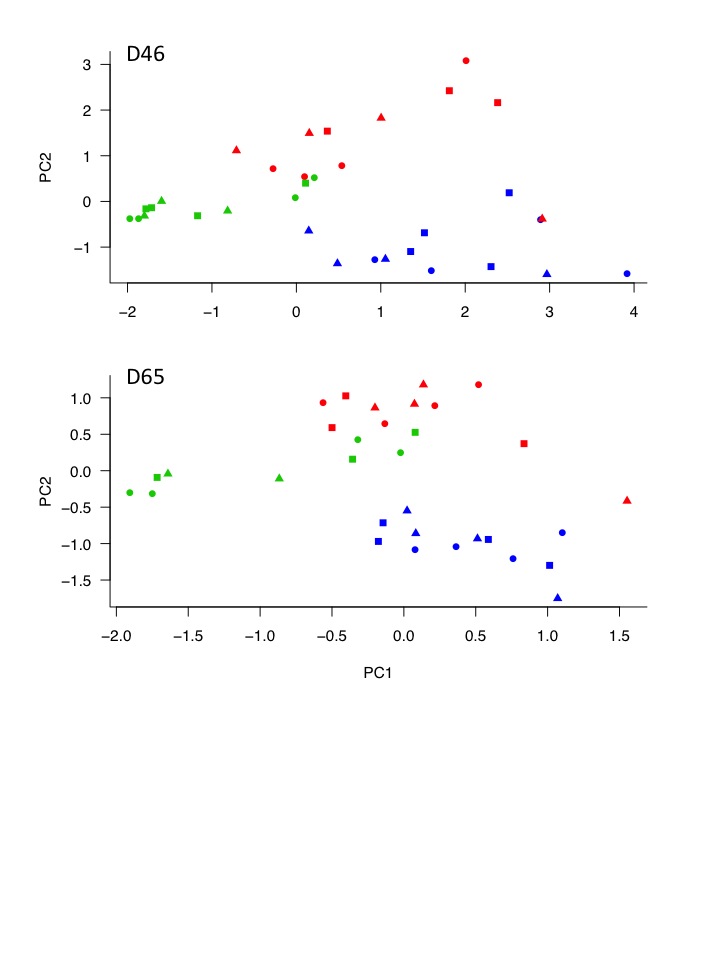
**Supplementary Figure 2.** The immune response depends on the primary strain. Scatterplot of the first and second principal components (PC1 and PC2) describes the antibody profiles of the 34 mice in the experiment. PC1 is a measure of the overall immune response; mice with high PC1 values have high titers of total, anti-Fla, anti-OspCA, anti-OspCK, and anti-OspCN IgG. PC2 is a measure of the specificity of the immune response; mice with high PC2 values have high titers of anti-OspCK IgG (and total IgG) but low titers of anti-OspCA IgG (and anti-Fla IgG). The top and bottom panels refer to the day 46 (D46) and day 65 (D65) of the blood samples, respectively. Points are colored by the primary strain (blue = strain A, red = strain K, green = strain N) and symbols refer to the secondary strain (circle = strain A; square = strain K; triangle = strain N). Points cluster by primary strain (color) but not by secondary strain (symbol).

**Supplementary Table 1.** Organs were infected by the primary inoculated strain, though strains did not infect at the same rate and some organs were more susceptible to infection, without interactions. (A) **Maximum likelihood estimates:** shown are the model structure (Inf = strain infected the organ; Order = order of inoculation (primary/secondary); Organ= Bladder, Ear skin, Heart, Mammary gland; Strain = strain identity), number of parameters (npar), deviance and the corrected AIC score (AICc). (B) **Log-likelihood ratio tests of the fixed factors of interest:** shown are the nested model comparisons, change in degrees of freedom (ΔDf), change in deviance (ΔDev), and p-value (p). (C) **Parameter estimates:** shown are the parameter estimates (contrasts and their standard errors) for the fixed factors of the best model.

| **A) Maximum Likelihood estimates** | | | | | | | | | | | | | |
| --- | --- | --- | --- | --- | --- | --- | --- | --- | --- | --- | --- | --- | --- |
| Rank | | Model | | | | npar | | | Deviance | | AICc | |  |
| 1  2  3  4  5 | Inf~Order+Organ+Strain  Inf~Order+Organ  Inf~Order+ Strain  Inf~Order*Organ*Strain  Inf~Organ+Strain | | | | | | 8  6  5  25  7 | | 174.4  190.8  200.2  163.3  332.8 | | 191.0  203.1  210.4  213.3  347.2 | |  |
| **B) Likelihood ratio test** | | | | | | | | | | | | | |
| Effect | | | Comparison | | | ΔDf | | ΔDev | | | | P | |
| All interactions  Strain  Organ  Order | | | 1 vs. 4  1 vs. 2  1 vs. 3  1 vs. 5 | | | 17  2  3  1 | | 11.13  16.38  25.78  158.3 | | | | 0.850  <0.001  <0.001  <0.001 | |
| **C) Parameter estimates of model 1** | | | | | | | | | | | | | |
| Variable | | | | Estimate | S.E. | | | Df | | z value | | | P |
| *Fixed effects* | | | |  |  | | |  | |  | | |  |
| Intercept | | | | 2.503 | 0.558 | | |  | | 4.48 | | |  |
| Strain  Strain K  Strain N  Organ  Ear skin  Heart  Mammary gland  Order of infection  Secondary | | | | -1.915  -1.376  -2.158  0.149  2.7.10-7  -4.800 | 0.525  0.513  0.567  0.546  0.542  0.581 | | | 1  1  1  1  1  1 | | -3.65  -2.68  -3.81  0.27  0.00  -8.27 | | | <0.001  0.007  <0.001  0.785  1  <0.001 |
| *Random effect (variance & std.dev)* | | | | |  | | |  | |  | | |  |
| Mouse | | | | 0 | 0 | | |  | |  | | |  |

**Supplementary Table 2.** Mouse-to-tick transmission of primary strain depended on the identity of the primary strain, the day of xenodiagnoses (age of infection), and the strain:day interaction. (A) **Maximum likelihood estimates:** shown are the model structure (Inf = tick infection status; D = day; S1 = primary strain; S2 = secondary strain), number of parameters (npar), deviance and the corrected AIC score (AICc). (B) **Log-likelihood ratio tests of the fixed factors of interest:** shown are the nested model comparisons, change in degrees of freedom (ΔDf), change in deviance (ΔDev), and p-value (p). (C) **Parameter estimates:** shown are the parameter estimates (contrasts and their standard errors) for the fixed factors of the best model.

| **A) Maximum Likelihood estimates** | | | | | | | | | | | | |  |
| --- | --- | --- | --- | --- | --- | --- | --- | --- | --- | --- | --- | --- | --- |
| Rank | | Model | | | | npar | | | Deviance | | AICc | | |
| 1  2  3  4  5 | Inf~D+S1+S1:D  Inf~D+S1  Inf~S1  Inf~D  Inf~D*S1*S2 | | | | | | 13  7  4  5  37 | | 261.2  252.2  294.9  306.5  308.5 | | 290.4  309.8  314.8  319.0  340.2 | | |
| **B) Likelihood ratio test** | | | | | | | | | | | | |  |
| Effect | | | Comparison | | | ΔDf | | ΔDev | | | P | |  |
| S2+S2interactions  S1:D  S1  D | | | Model 1 vs. Model 5 Model 1 vs. Model 2  Model 2 vs. Model 4  Model 2 vs. Model 3 | | | 24  6  2  3 | | 26.255  33.666  13.625  11.643 | | | 0.340  <0.001  0.001  0.009 | |  |
| **C) Parameter estimates of model 1** | | | | | | | | | | | | |  |
| Variable | | | | Estimate | S.E. | | | Df | | z value | | P |  |
| *Fixed effects* | | | |  |  | | |  | |  | |  |  |
| Intercept  Primary strain  Strain K  Strain N  Date  D30  D46  D65  Primary strain: Date  K: D30  N: D30  K: D46  N: D46  K: D65  N: D65 | | | | -0.144  0.332  -1.948  -2.313  -1.047  -0.919  0.702  0.975  1.101  2.355  0.993  0.223 | 0.357  0.521  0.573  0.294  0.294  0.320  0.450  0.526  0.422  0.489  0.465  0.591 | | | 1  1  1  1  1  1  1  1  1  1  1 | | -0.402  0.637  -3.402  -0.786  -3.559  -2.869  1.562  1.855  2.611  4.818  2.132  0.377 | | 0.524  <0.001  0.432  <0.001  0.004  0.118  0.064  0.009  <0.001  0.033  0.706 |  |
| *Random effect (variance & std.dev)* | | | | |  | | |  | |  | |  |  |
| Mouse | | | | 1.062 | 1.030 | | |  | |  | |  |  |

**Supplementary Table 3.** Among heterologous mice at D46 and D65, mouse-to-tick transmission depended on the order of inoculation (primary/secondary), strain identity (A, K, and N), the day of xenodiagnoses (age of infection), and the strain:day interaction. (A) **Maximum likelihood estimates:** shown are the model structure (Inf = tick infection status; D = day; O = order (primary/secondary); S = strain identity), number of parameters (npar), deviance and the corrected AIC score (AICc). (B) **Log-likelihood ratio tests of the fixed factors of interest:** shown are the nested model comparisons, change in degrees of freedom (ΔDf), change in deviance (ΔDev), and p-value (p). (C) **Parameter estimates:** shown are the parameter estimates (contrasts and their standard errors) for the fixed factors of the best model.

| **A) Maximum Likelihood estimates** | | | | | | | | | | |
| --- | --- | --- | --- | --- | --- | --- | --- | --- | --- | --- |
| Rank | Model | | | npar | | Deviance | AICc | | |  |
| 1 | Inf~O+S+D+S:D | | | 9 | | 503.3 | 521.5 | | |  |
| 2 | Inf~O+S+D | | | 7 | | 513.3 | 527.5 | | |  |
| 3 | Inf~O+S | | | 6 | | 518.8 | 530.9 | | |  |
| 4 | Inf~O*S*D | | | 14 | | 500.4 | 528.4 | | |  |
| 5 | Inf~O+D | | | 5 | | 530.0 | 540.1 | | |  |
| 6 | Inf~D+S | | | 6 | | 709.5 | 721.6 | | |  |
| **B) Likelihood ratio test** | | | | | | | | | | |
| Effect | | Comparison | | ΔDf | | ΔDev | | P | |  |
| O:S:D+O:S+O:D | | Model 1 vs. Model 4 | | 5 | | 2.91 | | 0.713 | |  |
| Strain:Date  Date  Strain  Order | | Model 1 vs. Model 2  Model 2 vs. Model 3  Model 2 vs. Model 5  Model 2 vs. Model 6 | | 2  1  2  1 | | 10.05  5.43  16.69  196.2 | | 0.007  0.020  <0.001  <0.001 | |  |
| **C) Parameter estimates of model 1** | | | | | | | | | | |
| Variable | | | Estimate | S.E. | D.F. | | Z value | | P | |
| *Fixed effects* | | |  |  |  | |  | |  | |
| Intercept | | | -1.180 | 0.432 |  | |  | |  | |
| Order of infection  Secondary strain | | | -4.110 | 0.480 | 1 | | -8.567 | | <0.001 | |
| Strain  Strain K  Strain N | | | 1.396  0.353 | 0.555  0.549 | 1  1 | | 2.514  0.642 | | 0.012  0.521 | |
| Date  D65 | | | -0.246 | 0.431 | 1 | | -0.570 | | 0.569 | |
| Strain:Date  K:D65  N:D65 | | | 0.294  -1.599 | 0.576  0.673 | 1  1 | | 0.511  -2.377 | | 0.609  0.017 | |
| *Random effect (variance & std.dev)* | | | |  |  | |  | |  | |
| Tick  Mouse | | | 0.00  1.21 | 0.00  1.10 |  | |  | |  | |

**Supplementary Table 4.** The titers of IgG were correlated. Above the diagonal are indicated the Pearson coefficients of the pairwise correlations, and below the diagonal are indicated the associated p-values.

|  | Total IgG | Fla-IgG | OspCA-IgG | OspCK-IgG | OspCN-IgG |
| --- | --- | --- | --- | --- | --- |
| Total IgG | - | 0.325 | 0.310 | 0.381 | 0.180 |
| Fla-IgG | <0.001 | - | 0.761 | 0.144 | 0.267 |
| OspCA-IgG | <0.001 | <0.001 | - | 0.088 | 0.273 |
| OspCK-IgG | <0.001 | 0.103 | 0.323 | - | 0.186 |
| OspCN-IgG | 0.041 | 0.002 | 0.002 | 0.035 | - |

**Supplementary Table 5.** The results from the principal component analysis (PCA). The first and second row show the % variance and the magnitude of the variance accounted for by the 5 principal components (PC1 to PC5). The five IgG variables were standardized to z-scores so the sum of the variances = 5*(1.0) = 5.0. Each column for rows 3 to 7 show the loadings of the five IgG variables for each principal component. The loadings facilitate the interpretation of the principal components.

|  | PC1 | PC2 | PC3 | PC4 | PC5 |
| --- | --- | --- | --- | --- | --- |
| % variance | 44.5 | 22.5 | 17.0 | 11.3 | 4.8 |
| variance | 2.220 | 1.124 | 0.846 | 0.563 | 0.240 |
| Total IgG | 0.43 | 0.43 | 0.34 | 0.72 | 0.01 |
| Fla-IgG | 0.56 | -0.35 | 0.16 | -0.21 | 0.70 |
| OspCA-IgG | 0.55 | -0.40 | 0.14 | -0.14 | -0.71 |
| OspCK-IgG | 0.29 | 0.73 | 0.05 | -0.62 | -0.06 |
| OspCN-IgG | 0.35 | 0.07 | -0.92 | 0.18 | 0.02 |

**Supplementary Table 6.** The strength of the immune response, summarized by PC1, depended on the day of blood sampling and the primary strain, but was independent of the secondary strain. (A) **Maximum likelihood estimates:** shown are the model structure (PC1= Principal Component 1; D = day; S1 = primary strain; S2 = secondary strain), number of parameters (npar), deviance and the corrected AIC score (AICc). (B) **Log-likelihood ratio tests of the fixed factors of interest:** shown are the nested model comparisons, change in degrees of freedom (ΔDf), change in deviance (ΔDev), and p-value (p). (C) **Parameter estimates:** shown are the parameter estimates (contrasts and their standard errors) for the fixed factors of the best model.

| **A) Maximum Likelihood estimates** | | | | | | | | | | | | |
| --- | --- | --- | --- | --- | --- | --- | --- | --- | --- | --- | --- | --- |
| Rank | | Model | | | | npar | | | Deviance | | AICc | |
| 1  2  3  4  5 | PC1~D+S1  PC1~S1  PC1~D+S1+S2  PC1~D+S1+S2+S1:D+S2:D  PC1~D | | | | | | 6  5  8  12  4 | | 119.3  125.1  118.5  113.9  150 | | 138.0  139.7  143.6  149.1  160.9 | |
| **B) Likelihood ratio test** | | | | | | | | | | | | |
| Effect | | | Comparison | | | ΔDf | | ΔDev | | | | P |
| S1:D+S2:D  S2  S1  D | | | Model 3 vs. Model 4  Model 1 vs. Model 3  Model 1 vs. Model 5  Model 1 vs. Model 2 | | | 4  2  2  1 | | 4.60  0.84  30.70  5.72 | | | | 0.33  0.66  <0.001  0.017 |
| **C) Parameter estimates of model 1** | | | | | | | | | | | | |
| Variable | | | | Estimate | S.E. | | | Df | | t value | | |
| *Fixed effects* | | | |  |  | | |  | |  | | |
| Intercept  Primary strain  Strain K  Strain N  Date  D65 | | | | 1.274  -0.517  -2.045  -0.594 | 0.256  0.296  0.318  0.234 | | | 1  1  1 | | 4.97  -1.75  -6.43  -2.53 | | |
| *Random effect (variance & std.dev)* | | | | |  | | |  | |  | | |
| Mouse | | | | 0.285 | 0.534 | | |  | |  | | |

**Supplementary Table 7.** The specific adaptive immune response, summarized by PC2, depended on the primary inoculated strain and the date considered, regardless of the seoncdary strain. (A) **Maximum likelihood estimates:** shown are the model structure (PC2= Principal Component 2; D = day; S1 = primary strain; S2 = secondary strain), number of parameters (npar), deviance and the corrected AIC score (AICc). (B) **Log-likelihood ratio tests of the fixed factors of interest:** shown are the nested model comparisons, change in degrees of freedom (ΔDf), change in deviance (ΔDev), and p-value (p). (C) **Parameter estimates:** shown are the parameter estimates (contrasts and their standard errors) for the fixed factors of the best model.

| **A) Maximum Likelihood estimates** | | | | | | | | | | | | | |
| --- | --- | --- | --- | --- | --- | --- | --- | --- | --- | --- | --- | --- | --- |
| Rank | | Model | | | | | npar | | Deviance | | AICc | |  |
| 1  2  3  4 | PC2~S1  PC2~D+S1+S2  PC2~D+S1+S2+S1:D+S2:D  PC2~1 | | | | | 5  8  12  1 | | | 78.8  76.7  74.5  131.8 | | 96.4  106.8  117.9  140.3 | |  |
| **B) Likelihood ratio test** | | | | | | | | | | | | | |
| Effect | | | Comparison | | | | ΔDf | ΔDev | | | | P | |
| S1:D+S2:D  S2+D  S1 | | | Model 3 vs. Model 1  Model 2 vs. Model 1  Model 1 vs. Model 4 | | | | 4  3  2 | 2.13  2.15  52.95 | | | | 0.711  0.542  <0.001 | |
| **C) Parameter estimates of model 1** | | | | | | | | | | | | | |
| Variable | | | | Estimate | S.E. | | | Df | | t value | | | P |
| *Fixed effects* | | | |  |  | | |  | |  | | |  |
| Intercept  Primary strain  Strain K  Strain N | | | | -0.994  1.958  1.018 | 0.145  0.205  0.208 | | | 1  1 | | 9.58  4.90 | | |  |
| *Random effect (variance & std.dev)* | | | | |  | | |  | |  | | |  |
| Mouse | | | | 0.00 | 0.00 | | |  | |  | | |  |
